# Textile-Based Triboelectric Nanogenerators for Wearable Self-Powered Microsystems

**DOI:** 10.3390/mi12020158

**Published:** 2021-02-05

**Authors:** Peng Huang, Dan-Liang Wen, Yu Qiu, Ming-Hong Yang, Cheng Tu, Hong-Sheng Zhong, Xiao-Sheng Zhang

**Affiliations:** School of Electronic Science and Engineering, University of Electronic Science and Technology of China, Chengdu 611731, China; huang_peng@std.uestc.edu.cn (P.H.); dlwen@std.uestc.edu.cn (D.-L.W.); qiuyu0420@163.com (Y.Q.); yangmhahu@163.com (M.-H.Y.); ctu@uestc.edu.cn (C.T.)

**Keywords:** triboelectric nanogenerators, nanogenerator, self-powered microsystems, textile

## Abstract

In recent years, wearable electronic devices have made considerable progress thanks to the rapid development of the Internet of Things. However, even though some of them have preliminarily achieved miniaturization and wearability, the drawbacks of frequent charging and physical rigidity of conventional lithium batteries, which are currently the most commonly used power source of wearable electronic devices, have become technical bottlenecks that need to be broken through urgently. In order to address the above challenges, the technology based on triboelectric effect, i.e., triboelectric nanogenerator (TENG), is proposed to harvest energy from ambient environment and considered as one of the most promising methods to integrate with functional electronic devices to form wearable self-powered microsystems. Benefited from excellent flexibility, high output performance, no materials limitation, and a quantitative relationship between environmental stimulation inputs and corresponding electrical outputs, TENGs present great advantages in wearable energy harvesting, active sensing, and driving actuators. Furthermore, combined with the superiorities of TENGs and fabrics, textile-based TENGs (T-TENGs) possess remarkable breathability and better non-planar surface adaptability, which are more conducive to the integrated wearable electronic devices and attract considerable attention. Herein, for the purpose of advancing the development of wearable electronic devices, this article reviews the recent development in materials for the construction of T-TENGs and methods for the enhancement of electrical output performance. More importantly, this article mainly focuses on the recent representative work, in which T-TENGs-based active sensors, T-TENGs-based self-driven actuators, and T-TENGs-based self-powered microsystems are studied. In addition, this paper summarizes the critical challenges and future opportunities of T-TENG-based wearable integrated microsystems.

## 1. Introduction

The rapid development of the Internet of Things technology has led to rapid growth in the number of smart wearable devices. It is estimated that the global shipment of smart wearable devices will reach 302.3 million in 2023 [1]. At the same time, with further research on electronic textile technology, smart textiles can be used as wearable clothing while also having the functionality and practicality of wearable electronic devices, which has attracted widespread attention in scientific research and commercial applications [2,3]. Generally, smart textiles realize the functions of wearable devices by embedding electronic components in the fabric [4,5]. The supply of energy is an indispensable part of smart fabrics and wearable devices. Most of the existing electronic devices are powered by batteries, but the batteries have shortcomings such as the need for recharging, short life, and large size, etc. Therefore, the supply of sustainable and effective clean energy is the key to solve the energy supply problem, which is of vital importance for smart fabrics and wearable devices. Energy harvesting from the environment and the human body is a current research hotspot. The existing solar cells [6,7], thermoelectric generators [8,9], and biofuel cells [10,11] are the main methods to harvest energy from the environment and human body, but they require external conditions such as sunlight, temperature, and auxiliary catalysts to work continuously and steadily.

**Figure 1:**
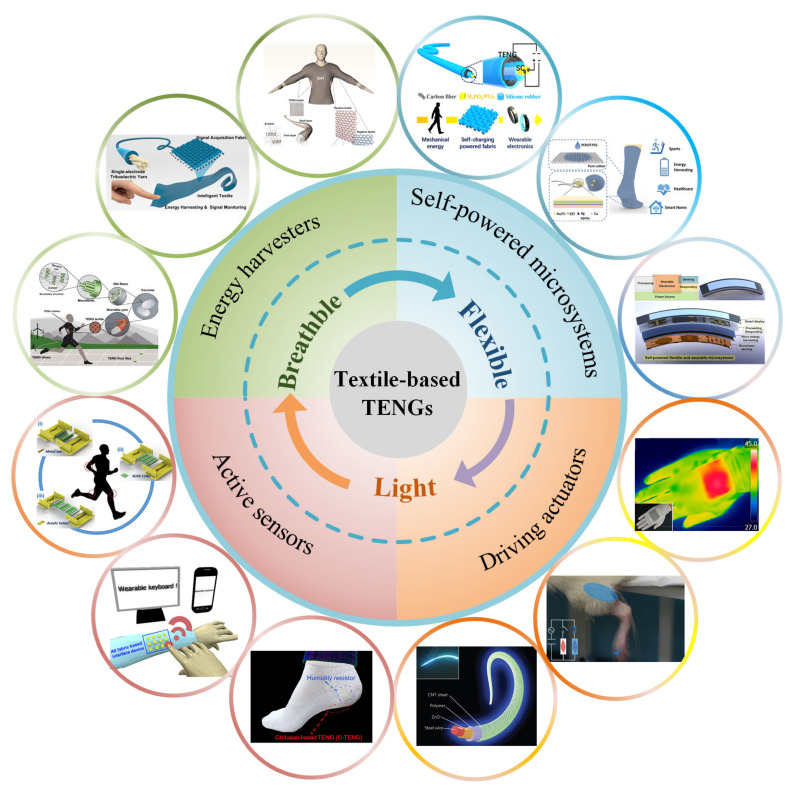
Self-powered microsystems based on textile-based triboelectric nanogenerators (T-TENG). T-TENG has many outstanding characteristics, including flexibility, breathability, and light weight, etc. It can form different T-TENGs in the form of fibers or fabrics, and the different weaving methods used can adapt to different situations. T-TENG can be used as energy harvesters, active sensors, and drive actuators, which can form microsystems. “Energy harvesters”. Reproduced with permission from Springer Nature (2017) [36]. Reproduced with permission from American Chemical Society (2020) [37]. “Active sensors”. Reproduced with permission from Elsevier (2018) [24]. Reproduced with permission from Elsevier (2018) [28]. Reproduced with permission from Elsevier (2020) [38]. “Driving actuators”. Reproduced with permission from American Chemical Society (2016) [39]. Reproduced with permission from Wiley (2019) [33]. Reproduced with permission from Springer Nature (2015) [40]. “Self-powered microsystems”. Reproduced with permission from American Chemical Society (2016) [41]. Reproduced with permission from American Chemical Society (2019) [42]. Reproduced with permission from Elsevier (2018) [43].

Triboelectric nanogenerator (TENG) has become a research hotspot after it was proposed by Wang [12] in 2012 because of its merits of wide material selection, simple production, and flexible and wearable characteristics [13,14]. Based on the coupling effect of triboelectrification and electrostatic induction, the TENG harvests ubiquitous mechanical energy in the natural environment, such as wind energy [15,16,17], water energy [18,19,20], and human body movements [21,22,23]. Based on the advantages of the above-mentioned TENG, scientists have discovered that textile-based TENG (T-TENG), which combines traditional textile technology with it, is a significant and promising field of the future development of wearable electronic products, due to the advantages of air permeability, flexibility, and flexible structure. As shown in Figure 1, T-TENG has been proven to be able to be used in sensing [24,25], energy harvesting [26,27], human-computer interaction [28,29], and many other aspects, which has very good prospects.

Some recent papers focus on the design and advancement of T-TENGs, which helps us to systematically understand the development of T-TENG and its optimization strategies. In addition to harvesting environmental energy, T-TENGs can also form active sensors [30,31], drive actuators [32,33], and constitute self-powered microsystems [34,35]. The textile-based self-powered microsystem is the final direction of T-TENG’s development, but there are only very limited reports. The research on the relationship among energy harvesters, sensors and actuators based on T-TENGs; the field of how to build T-TENG-based self-powered microsystems; and the future development trend of textile-based self-powered microsystems, are all very important. Because the successful development and mass production of T-TENG-based wearable electronic devices self-powered microsystems depend on further research on the relationship between energy harvesters, sensors, and actuators composed of T-TENGs.

Here, this article reviews the recent developments of T-TENGs and related technologies. First, we introduce the principle and working mode of TENGs. Next, the materials for manufacturing Textile-based TENGs, the method of improving the electrical output of T-TENGs, and the different structural design of T-TENGs as energy harvesters were illustrated. Meanwhile, we also elaborate the development process of T-TENG as a self-powered integrated microsystem, including sensors, actuators, and an integrated self-powered microsystem. Finally, we summarized the development of T-TENGs and prospected the challenges and opportunities T-TENGs face.

## 2. Progress of T-TENG

### 2.1. Working Mechanism

TENG works based on the triboelectric effect and electrostatic induction, when two materials with different electron affinities are in contact, they will get surface charges of different polarities, and then convert kinetic energy into electrical energy [44,45,46]. The triboelectric effect is a kind of contact electrification, when the surfaces of these two materials are in contact, electrons will transition from a high energy level to a low energy level. During the separation of the materials, the transitioned electrons cannot return completely and stay on the contact surface. The surface of the material with higher electron affinity is negatively charged, and the other surface is positively charged. When the external kinetic energy causes the two triboelectric materials to produce periodic motion, the induced potential difference between the electrodes also periodically changes. When the load is connected, periodic alternating current is generated, which can then be used as an energy harvester to convert kinetic energy into electrical energy [47,48,49,50,51,52].

**Figure 2:**
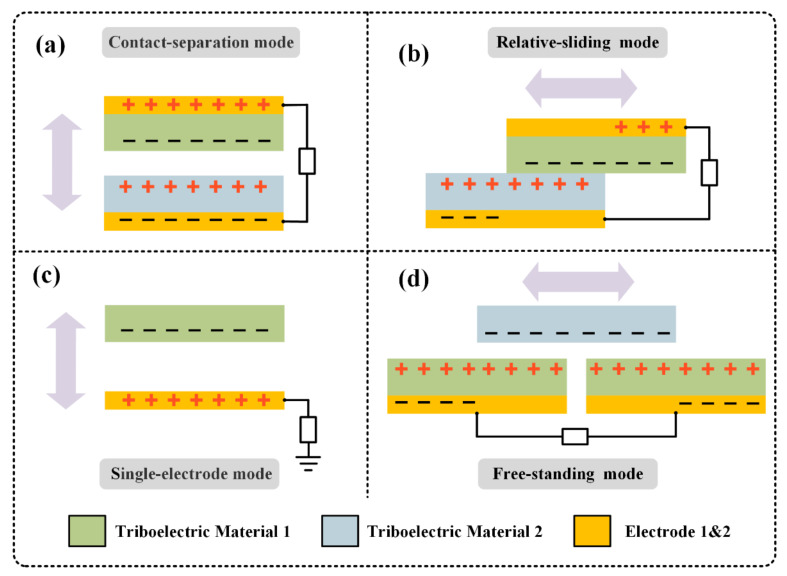
The four fundamental modes of the TENG. (**a**) Contact-separation (CS) mode. (**b**) Relative-sliding (RS) mode. (**c**) Single-electrode (SE) mode. (**d**) Free-standing (FS) mode.

Based on the basic principles of TENGs, four working modes have been developed: Contact-separation (CS) mode, relative-sliding (RS) mode, single-electrode (SE) mode, and free-standing (FS) mode [53,54,55], as shown in Figure 2. The CS mode uses polarization in the vertical direction, when the triboelectric pair contacts and separates in the vertical direction, due to the electrification of the contact, there is an induced potential between the triboelectric pairs, which then generates voltage and current (Figure 2a). CS mode is suitable for pressure, shock, vibration, and other environments. The TENG of the RS mode has a similar structure to the CS mode, and also uses the contact separation between two triboelectric pairs to generate voltage and current. The difference is that RS mode uses lateral polarization due to the relative sliding of the triboelectric pair (Figure 2b), which can work at a higher frequency due to its structural characteristics and improve efficiency. The SE mode has only one electrode and triboelectric layer, and the other triboelectric layer is an external object (Figure 2c), which simplifies the design of TENG. Since TENG in SE mode can remain stationary, it is usually designed as a touch sensor. The FS mode uses two fixed electrodes, and external objects can move freely between the electrodes to generate voltage and potential (Figure 2d). FS mode is suitable for detecting the movement of moving objects.

The working principle of T-TENG is the same as that of TENG. Most T-TENGs work in CS mode. For example, they can be placed inside the insole to collect the energy generated by human walking. In addition, T-TENG can also be embedded in the fabric. The pressure or pulling force generated by human body motion causes contact and separation between triboelectric materials, and T-TENG can collect energy. Based on the contact separation mode, T-TENG has been designed into various forms, such as yarn [56,57] and multi-layer fabric [58,59]. Since the freely moving dielectric does not require an electrical connection or electrode. T-TENG based on SE mode is also widely used for energy harvesting [60,61]. The SE mode T-TENG can be formed by a piece of fabric or a yarn with the human body, and the human skin is the conductor of another electrode. When the human body moves, there will be relative sliding between the fabrics, such as on the sleeves and under the arms. Two different friction surfaces can form the T-TENG of the RS mode. The fabric is usually designed as a linear-grating structure to improve the performance of this mode [62,63]. The FS mode is often combined with the CS mode. T-TENG in this mode is usually composed of three triboelectric materials. Freely moving triboelectric materials do not require additional electrodes [64,65].

### 2.2. Materials for T-TENG

#### 2.2.1. Fundamental Materials

The T-TENG mainly consists of three crucial components, i.e., fundamental materials, triboelectric materials, and electrodes. Fundamental materials, including fibers and their products, are usually used as substrates for the construction of T-TENG. The fundamental material properties are considered for substrates selection, such as air permeability, flexibility, stretchability, and weight. Fiber-based materials (e.g., yarns and fabrics) that are the fundamental materials of T-TENG can be divided into two types, natural and synthetic. Natural fibers are mainly divided into plant fibers (cotton, flax, abaca), animal fibers (wool, silk), and mineral fibers (asbestos). Synthetic fibers are fibers synthesized through chemical synthesis and mechanical processing from substances that do not contain natural fibers (coal, petroleum), such as polyester, nylon, and acrylic. Natural fibers are generally breathable, soft, and easily degradable, but they do not have as high mechanical strength as synthetic fibers. Synthetic fibers are generally durable, inexpensive, and fast-drying, but do not have good air permeability and degradability.

Yarn is made from short fibers through a series of processes (for example, the steps of making cotton yarn include opening, cleaning, blending, carding, carding, drafting, drafting, twisting, and winding). The yarn is made into the basic material of the fabric. Fabrics are mainly divided into woven fabrics, knitted fabrics and non-woven fabrics. Woven fabrics are interlocked and woven in horizontal and vertical directions by yarns. Knitted fabric is made of yarns that form loops. Non-woven fabrics directly convert fibers into fabrics through chemical and mechanical processes. Yarn, woven fabric, knitted fabric, and non-woven fabric can be used in T-TENG manufacturing.

#### 2.2.2. Triboelectric Materials

The primary rule of triboelectric materials selection is based on the relative difference in electron affinity, which can be explained as the intrinsic properties of gaining or losing electrons. In principle, when the relative difference of electron affinity between two triboelectric materials is greater, then the output performance of T-TENG is better. A standard method has been developed to quantify the electron affinity of different materials in 2019, which provided a very valuable reference for the selection of triboelectric materials in the T-TENG design process [66]. Triboelectric materials include most textile polymers and materials, which facilitate the production and application of T-TENG. For example, nylon, silk, polyethylene terephthalate (PET) polyurethane (PU), polylactic acid, etc. are usually used as the contact surface of TENG. Metals or metal particles (Au, Ag, Cu, etc.) are also commonly used as positive electrode materials for triboelectric, and they can also be used as electrode materials. In addition, materials such as polyvinylidene fluoride (PVDF), PTFE, polydimethylsiloxane (PDMS), and silicone rubber are also usually covered on fibers or fabrics to enhance the output of T-TENG. Zhang et al. demonstrated that silk protein is used as a triboelectric positive material [67], which has the characteristics of degradability, water solubility, and biocompatibility, and has a strong ability to lose electrons. Yao et al. reported TENG based on cellulose nanofibrils (CNF) [68]. These biodegradable CNFs and silk proteins are good candidates for wearable TENG. Guo et al. used pure polycaprolactone (PCL) electrospun nanofiber membrane as the electropositive triboelectric material, combined with polytetrafluoroethylene (ePTFE) as the electronegative triboelectric material, and constructed a highly efficient TENG [69]. No matter what material is used, T-TENG needs to have the characteristics of biocompatibility, flexibility, high mechanical strength, good air permeability, and high output.

#### 2.2.3. Electrode Materials

Choosing a suitable electrode material can improve the output efficiency of TENG. It serves as the conductive part of TENG. If the conductivity is not good, it will greatly reduce the output of TENG. Commonly used electrode materials can be divided into metal electrodes, carbon-based electrodes, and polymer electrodes.

Metals and their oxides such as copper, platinum, gold, aluminum, and indium tin oxide (ITO) can be used as electrodes, usually in sheet form as electrodes, which are not gas permeable and cannot be bent for a long time, so they are not suitable for T-TENG’s electrode. Another way is to use metal particles or metal nanowires as electrodes. Guo et al. use a dip coating method to dip nylon cloth into the silver nanowire solution to obtain a conductive fabric [39]. However, this method makes the metal electrode material easily fall off, the conductivity is reduced, and it is not friendly to the environment and the human body. On this basis, the use of polymer-wrapped conductive fiber core-shell devices can effectively avoid these problems [70]. In addition, conductive fabrics obtained by depositing metal on fabrics by sputtering, evaporation, and electroless plating can also be used as electrodes. For example, conductive fabric (Ni@fabric) has low cost, good flexibility, high mechanical strength, and good compatibility with flexible triboelectric materials.

As a degradable material, carbon-based materials are widely used as materials for TENG. Carbon-based electrodes such as carbon nanotubes (CNT) and graphene have the characteristics of good electrical conductivity and low cost, making them the best choice for TENG electrodes. Zhu et al. used graphene ink to cover the nylon surface to make a conductive fabric [71]. Souri et al. coated graphene nanosheets and carbon black (CB) on the yarn by ultrasonic treatment, and obtained a conductive and stretchable yarn [72]. Although carbon-based electrodes have many advantages, they cannot be widely promoted due to the difficulty of processing.

Conductive polymers have flexibility as a candidate material for the preparation of flexible TENG. Polystyrene sulfonate (PEDOT:PSS) is designed to exhibit inherent stretchability without elastomers, and is widely used as a conductor due to its high conductivity. Conductive polymer PEDOT:PSS has the characteristics of transparency and flexibility, and it has strong electron affinity. Wang et al. develop a TENG based on Ce-doped ZnO-PANI nanocomposite film [73]. In addition, materials such as polyacetylene (PA) and polypyrrole (PPy) are also used to make TENG electrodes.

Metal electrodes have good electrical conductivity, but they are easy to oxidize, and become unstable in a humid environment. Conductive polymers and carbon-based materials currently have lower electrical properties, but have good plasticity and degradability. Find Suitable T-TENG electrode materials are a current research focus.

### 2.3. Methods for the Performance Improvement of T-TENG

#### 2.3.1. Surface Modification

**Figure 3:**
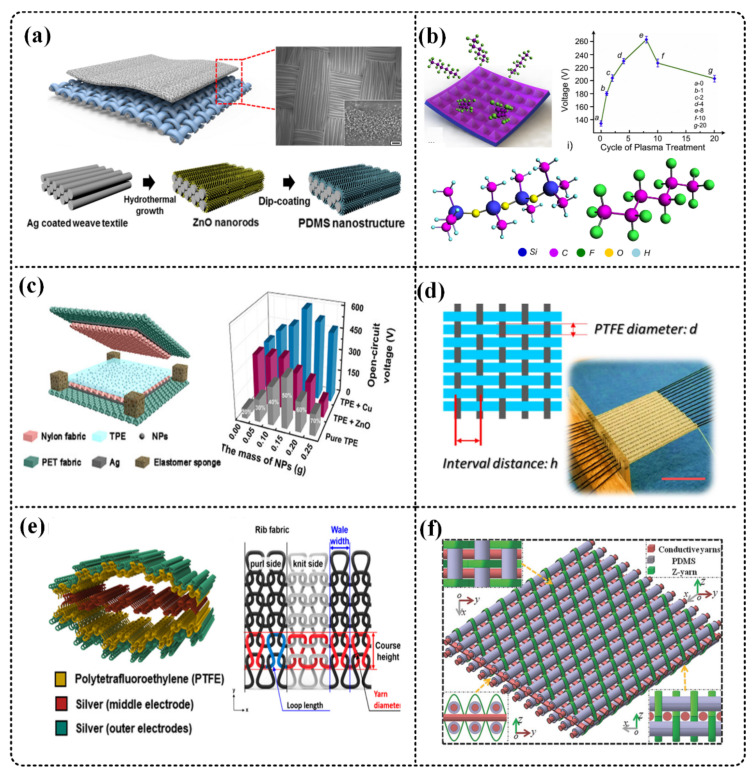
Methods to improve the electrical output of T-TENG. (**a**) Seung et al. perform nanopatterning on PDMS. Reproduced with permission from American Chemical Society (2015) [75]. (**b**) Zhang et al. used a single-step fluorocarbon plasma Table 2013. [79]. (**c**) Zhang et al. doped Cu nanoparticles into thermoplastic elastomer (TPE) composite fabrics, and the TENG made by them increased the output by 1.5 times. Reproduced with permission from American Chemical Society (2018) [81]. (**d**) Chen et al. studied the influence of the spacing between carbon wires and the line width of PTFE on the electrical output performance of TENG. Reproduced with permission from Elsevier (2018) [83]. (**e**) Kwak et al. used double-sided knitting and rib knitting to increase the electrical output of TENG. Reproduced with permission from American Chemical Society (2017) [84]. (**f**) Dong et al. used 3D structure design to improve TENG output performance. Reproduced with permission from Wiley (2017) [86].

One way of surface modification is to produce microstructures on the surface of the triboelectric material through micromachining to increase the contact area and increase the output of TENG, such as using photolithography templates [74], nano/micro processing technology [75,76], and ion beam etching [77] and other methods. Zhang et al. improved the output of TENG by making pyramid and V-shaped groove micro/nano structures on the surface of PDMS [78]. As shown in Figure 3a, Seung et al. applied PDMS on the ZnO nanorods of a silver-coated textile template and performed nanopatterning on PDMS [75], which increased the output voltage by four times.

Another way is to treat the triboelectric material by chemical modification or doping, so that the ability of the triboelectric material to gain or lose electrons is enhanced, thereby increasing the output. Zhang et al. proposed a simple and versatile technique, namely, a single-step fluorocarbon plasma to treat the surface of the TENG triboelectric layer (Figure 3b) [79], which improves the material’s vertical ionization energy. The output performance of the produced TENG increased by 278%. As shown in Figure 3c, Zhang et al. doped Cu nanoparticles into thermoplastic elastomer (TPE) composite fabrics, and the TENG made by them increased the output by 1.5 times [80]. Chu et al. used oxygen plasma to etch PDMS films to obtain nanostructures, and the use of SF_6_ plasma chemical modification, making the output current and voltage of TENG increase by more than 10 times [81].

#### 2.3.2. Structural Design

It is one of the commonly used methods to improve the output of TENG through structural design. T-TENG is made of fabric, so by changing the weaving method, the surface area ratio is increased, thereby improving the output performance of T-TENG. Weaving and knitting [82,83] are common knitting methods. In addition, 3D knitting [84,85] is also used to improve the output performance of T-TENG.

Pyo et al. used textiles composed of pile embroidery (rough texture) fibers as the contact surface to increase the effective triboelectric area, and compared with plain weave fibers, the output increased by 24 times [85]. As shown in Figure 3d, Chen et al. uses PTFE thread, carbon thread, and cotton thread to woven fabric TENG, and studied the influence of the spacing between carbon threads and the line width of PTFE on the electrical output performance of TENG. With the increase of PTFE line width, the output performance of TENG decreases [83].

TENG fabrics with 3D structures have also been gradually developed. As shown in Figure 3e, Kwak et al. used double-sided knitting and rib knitting to knit PTFE thread and silver thread into TENG. The selection of the knitted structure of the fabric highly boosted the generated total triboelectric charges and the output voltage by approximately 1170%, which is critical for realizing a high-performance wearable and stretchable TENG [84]. Dong et al. combined the stainless steel/polyester fiber blended yarn, the polydimethylsiloxane-coated energy-harvesting yarn, and nonconductive binding yarn, and used 3D structure design to produce a T-TENG (Figure 3f) [86], the maximum peak power of 3D textiles. The density can reach 263.36 mW/m^2^ at a percussion frequency of 3 Hz, which is several times that of traditional 2D textile TENG.

## 3. T-TENGs for Wearable Self-Powered Microsystems

### 3.1. T-TENG Configurations: Fiber and Fabric

#### 3.1.1. Fiber-Based TENGs

There are more and more applications in T-TENG based on yarn structure or core-shell structure [23,26,27,87], usually each yarn has a separate electrode and triboelectric material, so it can be used as a TENG alone for energy collection, TENG fabric can also be obtained by weaving.

**Figure 4:**
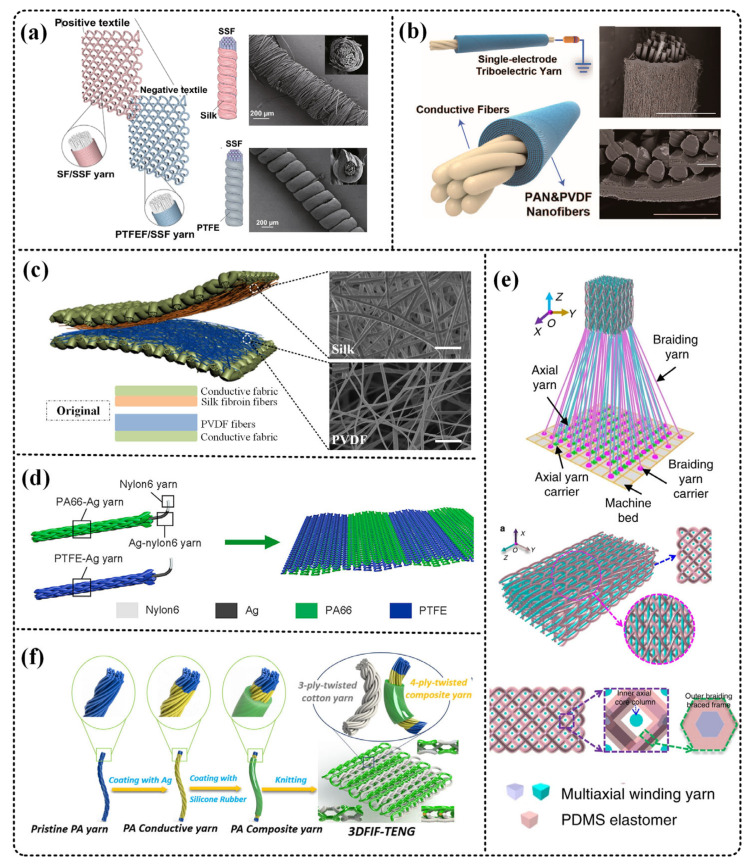
Different T-TENGs as energy harvesters. (**a**–**c**) Fiber-based TENGs. (**d**–**f**) Fabric-based TENGs. (**a**) Ye et al. manufactured silk fiber (SF), polytetrafluoroethylene fiber (PTFEF), and stainless steel fiber (SSF) into TENG yarn with a core-shell structure. Reproduced with permission from Springer Nature (2017) [36]. (**b**) Ma et al. produced a single-electrode triboelectric yarn with a spiral core-shell structure. The inner layer uses conductive silver nanowires, and the outer layer uses polyvinylidene fluoride (PVDF) and polyacrylonitrile (PAN) hybrid nanofibers. Reproduced with permission from American Chemical Society (2020) [37]. (**c**) Guo et al. electrostatically spun silk fibroin and polyvinylidene fluoride nanofibers onto conductive fabrics to manufacture a full-fiber hybrid piezoelectric-reinforced triboelectric nanogenerator. Reproduced with permission from Elsevier (2018) [90]. (**d**) Dong et al. used silver yarn wrapped with nylon and PTFE threads for knitting, and obtained fabric-based TENG. Reproduced with permission from Elsevier (2020) [92]. (**e**) Dong et al. designed a stable and soft 3D TENG fabric with the aid of a three-dimensional five-way weave structure. Reproduced with permission from Springer Nature (2017) [93]. (**f**) Chen et al. used double-needle flat knitting technology to design a TENG with 3D double-sided interlock. Reproduced with permission from Elsevier (2020) [38].

In 2019, Ye et al. adopted a unique layered structure design to make silk fiber (SF), polytetrafluoroethylene fiber (PTFEF), and stainless steel fiber (SSF) into TENG yarn with a core-shell structure [36]. As shown in Figure 4a, the positive triboelectric fabric is wrapped with SF, and the negative triboelectric fabric is composed of PTFEF and SSF. The output of TENG made from these yarns reaches 3.5 mW/m^2^ and after 2.3 million cycles of deformation, electrical. The output does not drop significantly and has good stability. Finally, the possible application prospects in human-computer interaction and motion tracking are demonstrated.

In 2020, Ma et al. used electrospinning technology to manufacture a single-electrode triboelectric yarn with a spiral core-shell structure (Figure 4b) [37]. The inner layer uses conductive silver nanowires, and the outer layer uses polyvinylidene fluoride (PVDF) and polyacrylonitrile (PAN) hybrid nanofibers. The yarn has the characteristics of small diameter (350.66 um), ultra-light (0.33 mg/cm), and high output (40.8 V, 0.705 μAc/m^2^, 2.5 Hz). In addition, the plain weave fabric composed of this yarn can monitor the tiny movements of the human body or insects.

In addition to TENG based on the core-shell structure, TENGs based on the sandwiched fiber structure have also been widely used [88,89]. Usually, the yarn or fiber is sandwiched between the material layers, and the fiber or yarn used as a triboelectric pair may not have durability. We can also rely on the outermost material to improve protection, such as conductive fabric as an electrode. Guo et al. electrospun silk protein and polyvinylidene fluoride nanofibers onto a conductive fabric to manufacture a full-fiber hybrid piezoelectric-enhanced triboelectric nanogenerator [90]. As shown in Figure 4c, the output power density of the hybrid nanogenerator reached 310 μW/cm^2^, and has good air permeability and flexibility, and has demonstrated its application in sports and fall detection.

#### 3.1.2. Fabric-Based TENGs

TENG based on single yarn or sandwich structure is mostly used for contact separation in the vertical direction. However, in daily life, the human body generates various types of mechanical energy, including lateral triboelectric between clothes or between clothes and skin caused by mechanical movements such as arm swings and walking. Therefore, the use of fabric-based TENG can better collect mechanical energy, and fabric-based TENG can be better integrated on clothing. Various patterns of fabric TENG have now been developed to collect the mechanical energy generated by the human body [91,92].

Dong et al. used silver yarn wrapped with nylon and PTFE threads for knitting, and obtained fabric-based TENG (Figure 4d). Knitted pants made of this fabric can be used to collect energy from knee bending. In the compression exercise mode, the output has reached 7531 μW/m^2^. It is worth noting that the fabric adopts a double-layer cross-woven structure, which can be seamlessly woven into clothes [92].

3D weaving technology has also been gradually applied to the manufacture of T-TENG, and it has been proven that it can increase the output of TENG [38,93]. Dong, et al. designed a stable and soft 3D TENG fabric with the aid of a three-dimensional five-way weaving structure [93]. As shown in Figure 4e, the TENG has a good compression resilience due to the space frame column structure formed between the outer braided yarn and the inner shaft yarn, which increases the output of the TENG. Finally, applications such as wireless motion monitoring and multi-functional man-machine interface were demonstrated, showing good application prospects.

Chen et al. used the double-needle flat knitting machine technology to design a 3D double-sided interlocking TENG (Figure 4f) [38]. The TENG can be used for stretch sensing, tactile sensing, and other sensing. The combination of interlocking and weft knitting makes this fabric more flexible. Substrate-free and 3D structural design may provide a promising direction for self-powered, stretchable, wearable devices.

### 3.2. T-TENG Sensing Function: Active Sensors

In addition to the TENG used to harvest energy for the self-powered microsystem, it can also be used as a sensor and actuator. The biggest advantage of TENG as a sensor is that it can output different electrical signals with external changes and does not require external power supply. Such sensors are also called active sensors. TENG as an active sensor has been proven to be used for pressure, stretch, humidity, touch, and other detection. It has good application prospects.

**Figure 5:**
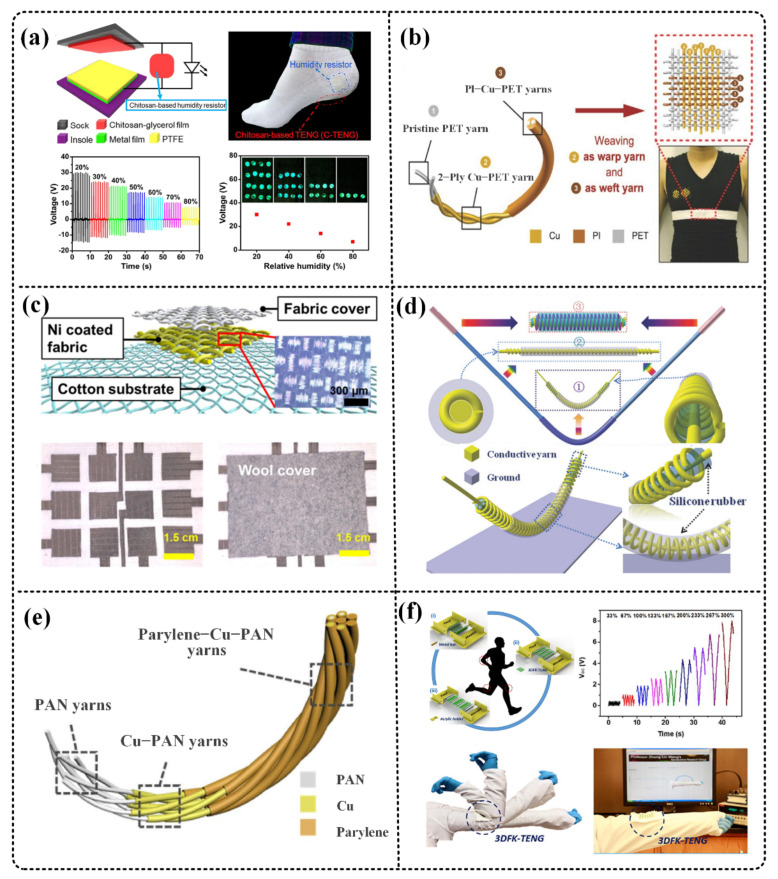
T-TENG acts as active sensors. (**a**) Jao et al. developed a TENG based on chitosan, which has different voltage outputs under different humidity environments. Reproduced with permission from Elsevier (2018) [24]. (**b**) Zhao et al. designed a T-TENG based on polyethylene terephthalate (PET) and copper Cu to monitor human respiratory rate and depth. Reproduced with permission from Wiley (2016) [25]. (**c**) Jeon et al. designed a TENG-based wearable fabric keyboard completely using commercial fabric. Reproduced with permission from Elsevier (2018) [28]. (**d**) Dong et al. designed a coaxial spring-like spiral-wound structure of TENG as a skipping rope counter. Reproduced with permission from Wiley (2018) [29]. (**e**) Zhao et al. covered the polyacrylonitrile yarn with copper (Cu) and parylene to obtain a composite yarn, which was then woven into TENG pressure sensors with different structures. Reproduced with permission from Elsevier (2020) [30]. (**f**) The 3D double-sided interlocking fabric TENG designed by Chen et al. can be used for tensile testing. Reproduced with permission from Elsevier (2020) [38].

Physiological signal monitoring is one of the important functions of smart fabrics. Jao et al. developed a TENG sensor based on chitosan. As shown in Figure 5a, the TENG has different voltage outputs in different humidity environments and can be used for humidity detection. In addition, it can also be used for gait detection and sweat detection, with versatility. When the TENG is used as an energy harvester, there is no significant change in the electrical output performance of the TENG within the relative humidity range of 20–80% [24]. In addition, Zhao et al. designed a T-TENG based on PET and copper Cu. The yarns are criss-crossed to generate triboelectric charges. The short-circuit current density is 15.50 mA/m^2^, and the TENG is integrated into the chest strap (Figure 5b) to monitor the human breathing frequency and depth [25].

Motion detection such as pressure, stretch, and touch is also an important function of smart fabrics. Jeon et al. designed a TENG-based wearable fabric keyboard (Figure 5c). The 12-unit keyboard is completely made of commercial fabrics, and has the advantages of foldability and washability. Finally, the keyboard is used to verify the keyboard operation by inputting words and playing music. Shows the application of TENG in touch sensing [28]. Dong et al. designed a coaxial spring-like spiral wound structure of TENG (Figure 5d), which has excellent mechanical properties. At a fixed frequency of 3 Hz, the maximum average power density of a single yarn TENG can reach 11 and 0.88 W/m^3^ in compression and tension, respectively. It also demonstrated its application as a skipping rope counter, self-powered gesture recognition, and golf scoring system [29].

Zhao et al. covered the polyacrylonitrile yarn with copper (Cu) and parylene to obtain a composite yarn, and used this yarn to knit into a TENG pressure sensor of different structures (Figure 5e), the pressure sensor is in the range of 0–25 kPa. There is a good response inside, the highest sensitivity reaches 0.344 V/kPa (less than 0.25 kPa), and it can be washed with good air permeability [30]. In addition, the 3D double-sided interlocking fabric TENG designed by Chen and others can be used in the stretch test (Figure 5f), the maximum stretch range can reach 300%. When stretching with different lengths, TENG will output different voltages. In addition, it can also be used for touch and pressure detection. The voltage output under a pressure of 0.4 kPa to 4 kPa shows a good linear correlation [38].

### 3.3. T-TENG Powering Function: Driving Actuators

Collecting energy and monitoring environmental factors are the primary tasks of self-powered microsystems. What is more important is to respond to changes. Due to the instantaneous high voltage generated by TENG, it can directly drive the micro-drive, or store the power in a capacitor or battery, and indirectly drive the micro-drive. This section gives examples of actuators that can be directly or indirectly driven by TENG, including heating fabrics, biological actuators, fiber actuators, and fiber light emitting devices.

**Figure 6:**
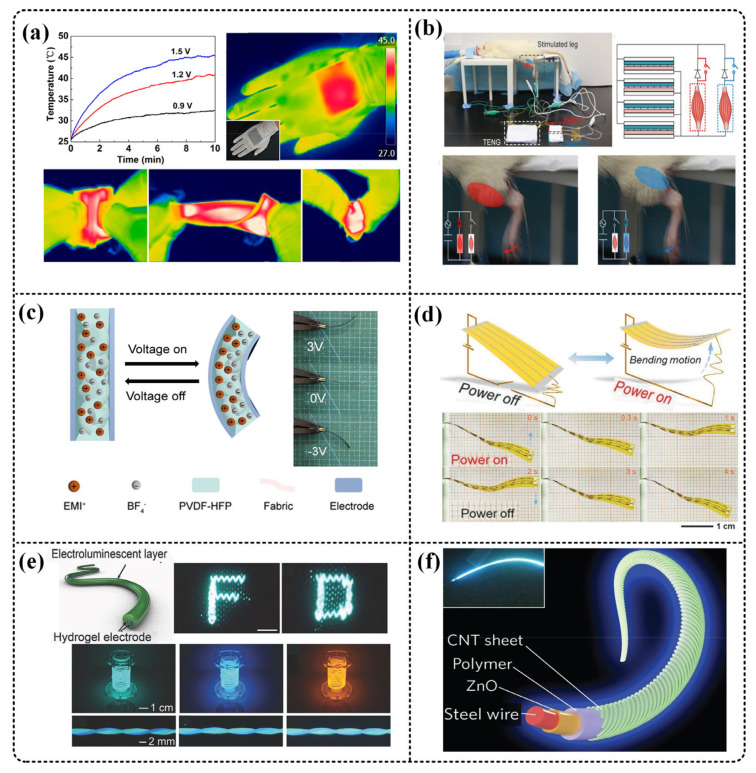
T-TENG is used to drive the actuators. (**a**) Guo et al. coated fluoroalkylsilane (FAS), polydimethylsiloxane (PDMS), and silver nanowires (AgNWs) on nylon cloth by dip coating to obtain a fabric that can be heated. Reproduced with permission from American Chemical Society (2016) [39]. (**b**) He et al. proposed a diode-enhanced T-TENG and used this TENG to stimulate the tibialis anterior and gastrocnemius muscles of anesthetized mice. Reproduced with permission from Wiley (2019) [33]. (**c**) Wu et al. developed a fabric actuator based on carbonized products and PEDOT:PSS electrodes. [94]. (**d**) Chen et al. woven conductive fibers into polymer tapes to prepare actuators, using spring-like CNT fibers to prepare actuators. Reproduced with permission from Wiley (2015) [95]. (**e**) Zhang et al. also produced a super stretchable luminescent fiber. Reproduced with permission from Wiley (2018) [96]. (**f**) Zhang et al. developed a color-tunable fibrous polymer light-emitting device using a coaxial structure. Reproduced with permission from Springer Nature (2015) [40].

One of the functions of fabric is to keep warm. Therefore, fabrics with heating function are one of the development directions of smart fabrics. Zhang et al. used an improved reactive vapor deposition method to coat PEDOT material on the fabric to obtain a heatable fabric. It is demonstrated that the temperature rises from the ambient temperature (19 °C) to the equilibrium temperature of 56 °C within 20 s when the heating fabric is powered by a 4.5 V alkaline battery. In addition, the fabric balance temperature is adjustable, soft, and breathable [32]. Guo et al. coated fluoroalkyl silane (FAS), PDMS, and silver nanowires (AgNWs) on nylon cloth by dipping to obtain a TENG fabric for energy. It can be insulated and heated while collecting (Figure 6a). The fabric TENG can be heated from 25 °C to 45 °C (within 4 min) at a low voltage of 1.5 V, showing an example of TENG and heater integration [39].

Old people’s athletic ability declines, and some diseases (stroke and Parkinson’s disease) require further rehabilitation by stimulating muscles or nerves. He et al. proposed a diode-enhanced T-TENG and used this TENG to stimulate the tibialis anterior and gastrocnemius muscles of anesthetized mice (Figure 6b). The results show that within the test range, the current has a linear relationship with the force output by the mouse’s hind legs. It is worth noting that this work integrates T-TENG and high-voltage diodes for the first time to form a higher open circuit voltage [33]. Zhang et al. also showed that TENG stimulates the sciatic nerve of a frog to control its leg movement [79], which shows the application of TENG in the biological field.

In addition, textile-based actuators have gradually been developed [94,95]. Wu et al. developed a fabric actuator based on carbonized products and PEDOT:PSS electrodes, using ion etching technology to make the surface of the fabric hydrophilic, so that the conductive ink is directly formed on the surface of the fabric (Figure 6c). The actuator can produce a strain difference of 0.28% under a voltage of 3 V, and a strain rate of 2.8%/s at 10 Hz [94]. Chen et al. woven conductive fibers into a polymer tape to prepare the actuator (Figure 6d) using a spring Actuators made of CNT fibers show excellent electromagnetic drive performance superior to single-layer CNT fibers. The actuator can be driven by low voltage (<10 V/cm), and the highly reversible programmable drive includes bending, contraction, extension, and rotation, and still maintain good performance after thousands of actions [95].

LEDs can produce light of different colors and different intensities, and we can directly obtain important information transmitted by light through our eyes. Textile-based light-emitting devices have also been gradually developed [40,96,97,98]. Zhang et al. produced a super-stretched light-emitting fiber (Figure 6e), and a polymer hydrogel of polyvinyl alcohol and polyethylene oxide was used for internal conductive electrodes. The maximum stretching degree can reach 800%, and the optical fiber brightness can be completely restored under 300% strain. The application of communication in the brain interface was demonstrated, showing a good application prospect [96]. In addition, Zhang et al. also developed a color-tunable fibrous polymer light-emitting device. (Figure 6f). Using the same shaft structure includes a modified metal cathode wire and a conductively arranged CNT sheet anode. The device has the same brightness in all directions. By assembling two light-emitting devices of different colors, different colors can be realized, and the current can be changed to control the light-emitting brightness [40].

### 3.4. T-TENG Integrating Function: Self-Powered Microsystems

The textile-based flexible self-powered integrated micro system is the ultimate vision of smart fabrics. Due to the limitations of technology and materials, textile-based self-powered integrated microsystems still need some time to develop. However, now there are some integrated systems, such as energy harvesting and energy storage, such as TENG and super capacitor integration [34,41,73,99,100,101], TENG and battery integration [35,102], the integration of energy harvesting and sensing [42,103], hybrid energy harvesting [104], etc., that show great application prospects.

**Figure 7:**
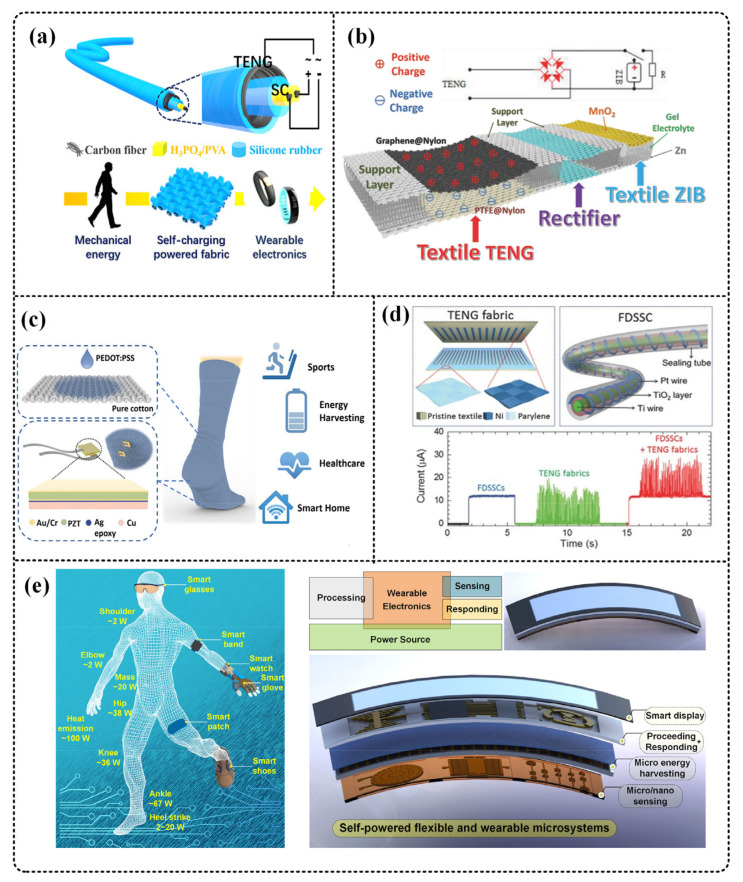
T-TENG serves as the basis for integrated microsystems. (**a**) Yang et al. used a coaxial structure to integrate TENG and SC into one fiber. Reproduced with permission from American Chemical Society (2016) [41]. (**b**) Wang et al. used 3D structure to integrate TENG, rectifier bridge, and Zn ion battery in a fabric. Reproduced with permission from Wiley (2018) [102]. (**c**) Zhu et al. mixed and integrated PEDOT:PSS-coated fabric TENG and lead zirconate titanate (PZT) piezoelectric chips. It can realize multi-sensing and energy harvesting. Reproduced with permission from American Chemical Society (2019) [42]. (**d**) Pu et al. integrated the fabric TENG with a grating structure and the fibrous dye-sensitized solar cell (FDSSC) into a cloth. Reproduced with permission from Wiley (2016) [104]. (**e**) All-in-one self-powered flexible microsystems proposed by Zhang et al. Reproduced with permission from Elsevier (2018) [43].

Supercapacitors are a potential energy storage technology, which is widely used because of its stability, safety, and flexibility. Yang et al. used a coaxial structure to integrate TENG and SC in a single fiber (Figure 7a) which can collect mechanical energy while storing energy in the fiber. A supercapacitor with a specific capacitance of 31.25 mF/g is made inside the fiber. At the same time, the maximum output power of TENG outside the fiber is 1.12 μW, which realized the integration of energy collection and storage [41].

The energy density of batteries is greater than that of supercapacitors, and they have been used in many electronic products such as smartphones, tablet computers, and electric cars. Wang et al. used 3D structure to integrate TENG, rectifier bridge, and Zn ion battery in a piece of fabric (Figure 7b). The energy collected by TENG can be directly stored in the battery through the rectifier circuit. The maximum output power of TENG is about 18.19 mW/m^2^, and the maximum specific capacity of the flexible ZIB is about 265 mAh/g, and it is demonstrated by powering electronic watches [102].

Zhu et al. mixed and integrated PEDOT:PSS-coated fabric TENG and lead zirconate titanate (PZT) piezoelectric chips (Figure 7c). A TENG sock can output 1.7 mW of power at a frequency of 2 Hz, collecting energy while monitoring physiological information such as gait, contact force, and sweat level [42].

Hybrid energy harvesters are also the current development trend. It can effectively improve energy collection efficiency and overcome the shortcomings of single energy collection. Pu et al. used a laser scratch mask and electroless deposition (ELD) nickel plating methods to develop a fabric TENG with a grating structure (Figure 7d) and integrated this TENG and fibrous dye-sensitized solar cell (FDSSC) in a piece of cloth, the TENG output reaches 3.2 W/m^2^ (at a speed of 0.75 m/s) at AM 1.5 solar energy (100 mW/cm^2^), while the average power conversion efficiency of FDSSC is 6%. It has realized the collection of human energy while collecting sunlight [104]. In addition, Zhang et al. proposed the concept of an all-in-one self-powered microsystem [43]. As shown in Figure 7e, it can harvest the energy generated by the human body and use the energy to power the micro system to sense the corresponding environmental and biological changes, which shows a huge application prospect.

The above example shows a multifunctional smart fabric based on TENG fabric. Although only part of the function integration is realized, it does not affect our prediction of the development of smart fabrics. The future self-powered microsystem based on fabric TENG might be highly integrated, multifunctional, flexible, and wearable. Energy harvesters, energy storage units, sensors, actuators, etc., are integrated on a fabric. The energy harvester collects one or more kinds of energy, and converts and stores it through an energy storage unit. The output DC can be supplied to sensors and microprocessors. In particular, TENG itself can respond to changes in different environments, output different electrical signals, and can be used as an active sensor. Finally, the actuator can react accordingly. Therefore, researchers proposed a concept of “all-in-one” self-powered smart microsystems by integrating TENGs to discrete components, including, but not limited to, sensors, actuators, signal proceeding circuits, and power management circuits [43]. This integrated self-powered microsystem can not only perceive the external environment, but also perceive human body information. This device converts the energy produced by the human body or the environment to electricity to power itself, thus it works independently and autonomously. It can respond accordingly to changes in the external environment. An “all-in-one” textile-based self-powered integrated microsystem is one of the future development directions, which will be widely used in many fields such as wearable electronic equipment, biomedical testing, and the Internet of Things.

## 4. Conclusions and Outlooks

TENG is considered to be one of the most promising candidates for power supply for next-generation wearable devices due to its advantages like flexibility, simple production, and wide selection of materials. While T-TENG has the characteristics of air permeability, flexibility, and wearability, which is an important development direction of TENG. In recent years, scientists have devoted themselves to constructing T-TENG energy harvesters, active sensors, and actuators, etc., and combining them to form a textile-based self-powered microsystem.

Herein, this article reviews the recent developments of T-TENG and related technologies. The selection of T-TENG fabric materials is very wide, and commonly used textile materials such as cotton, linen, nylon, etc. can be used as basic fibers to fabricate. By coating metal, carbon-based conductive materials, and polymer conductive materials on fibers or fabrics, the fibers and fabrics are conductive, which can be used as electrodes for T-TENG. Textile materials such as cotton and silk can be used as triboelectric materials. In addition, polymer materials and metal materials with a strong ability to gain or lose electrons, and degradable materials such as silk protein, are also widely used as triboelectric materials. As the output of TENG is usually not very high, scientists have discovered through research that physical methods such as etching can increase the surface microstructure or chemical methods such as fluorocarbon plasma treatment and chemical doping to increase the output of TENG. Core-shell structure, sandwich structure, and fabric structure are the three basic structures of T-TENG. The use of different knitting (such as knitting and 3D knitting) and arrangement structure is also one of the important and efficient methods to improve the electrical output of T-TENG. Most T-TENG works in vertical separation mode, which can be used as active sensors such as touch, pressure, and stretch, and does not require external power supply. As an energy harvester, TENG can supply power to fabric actuators. Textile-based heaters, actuators, biological actuators, and light-emitting devices have been developed. Multifunctional integrated smart fabrics are one of the future directions. T-TENG-based energy harvesting-storage units, energy harvesting-sensing units, and hybrid energy harvesting units have been developed. As society’s demand for wearable devices increases, multi-functional integrated smart fabrics are one of the significant future directions. T-TENG-based energy harvesting-storage units, energy harvesting-sensing units, and hybrid energy harvesting units have been developed and they show the great potential of textile-based self-powered microsystems. Finally, we have made an outlook for the future of textile-based self-powered microsystems, which should have many characteristics such as high integration, multi-functionality, air permeability, and flexibility.

Although smart fabrics based on T-TENG are developing rapidly, they still face many challenges. This review proposes that further research should be carried out in the following aspects. Firstly, compared with the traditional thin film or rigid TENG, the output of T-TENG is usually lower. How to improve the output of T-TENG is the current research hotspot. Although it has been proven that the existing TENG exhibits high peak power, it is not very simple to judge the actual output power, and TENG has the characteristics of high output voltage, low output current, high impedance, etc., and power management circuits are usually added to improve TENG’s output efficiency. How to integrate power management circuits on the fabric is also a challenge. Secondly, the T-TENG needs to be integrated into clothing eventually, which requires it to be washable, and have good air permeability and flexibility. It is also necessary to overcome the influence of humidity, temperature, PH, and other instabilities caused by changes in human environment on the output performance of T-TENG. Although many T-TENGs have been proven to have washability, stretchability, etc., there is currently no unified evaluation system for fiber electronic equipment. It is very important to develop T-TENG that can work in complex environments. Thirdly, safety is very important for actual use. Existing electrodes and friction materials such as heavy metals can cause harm to the human body and the environment, and finding suitable safe degradable materials is also crucial. For integrated electronic fabrics, safety issues such as the battery and capacitor of the energy storage unit during work and storage must also be considered. Fourthly, for the practical applications of wearable devices that are not powered by power, they need to be able to be mass-produced, and at the same time, they need low-cost materials and mature manufacturing processes. The existing complex manufacturing processes and expensive materials are also wearable self-powered device development issues that need to be resolved. Fifthly, it is a critical issue for T-TENGs to further improve their robustness and reliability. Noncontact mode, rolling structural, liquid-solid contact, self-recovery, and encapsulated TENGs have been developed, which are five feasible strategies to improve robustness and stability [105]. The developed strategies can make TENG work stably in complex environments to a certain extent, but each strategy performs different functions, so further applications are limited. The development of T-TENGs that are suitable for a variety of harsh environments is the future trend. Finally, although there are now some electronic devices based on textiles, certain fiber-based electronic devices, such as transistors and memories, are still in the research stage and are far from practical. Currently, electronic components can only be discretely integrated on fabrics. Further exploration is needed to realize a real fabric microsystem.

The development of T-TENG electronic products requires the joint efforts of professionals in different fields. Although the T-TENG microsystem is still in the development stage and is facing huge challenges, it has shown great potential and attractive prospects in wearable electronics, biomedicine, and the Internet of Things, which deserves further research.

## Data Availability

Data is contained within the article.
